# Probing the Role of Protein Surface Charge in the Activation of PrfA, the Central Regulator of *Listeria monocytogenes* Pathogenesis

**DOI:** 10.1371/journal.pone.0023502

**Published:** 2011-08-12

**Authors:** Bobbi Xayarath, Karl W. Volz, Jennifer I. Smart, Nancy E. Freitag

**Affiliations:** Department of Microbiology and Immunology, University of Illinois at Chicago, Chicago, Illinois, United States of America; Baylor College of Medicine, United States of America

## Abstract

*Listeria monocytogenes* is a food-borne intracellular bacterial pathogen capable of causing serious human disease. *L. monocytogenes* survival within mammalian cells depends upon the synthesis of a number of secreted virulence factors whose expression is regulated by the transcriptional activator PrfA. PrfA becomes activated following bacterial entry into host cells where it induces the expression of gene products required for bacterial spread to adjacent cells. Activation of PrfA appears to occur via the binding of a small molecule cofactor whose identity remains unknown. Electrostatic modeling of the predicted PrfA cofactor binding pocket revealed a highly positively charged region with two lysine residues, K64 and K122, located at the edge of the pocket and another (K130) located deep within the interior. Mutational analysis of these residues indicated that K64 and K122 contribute to intracellular activation of PrfA, whereas a K130 substitution abolished protein activity. The requirement of K64 and K122 for intracellular PrfA activation could be bypassed via the introduction of the *prfA* G145S mutation that constitutively activates PrfA in the absence of cofactor binding. Our data indicate that the positive charge of the PrfA binding pocket contributes to intracellular activation of PrfA, presumably by facilitating binding of an anionic cofactor.

## Introduction

*L. monocytogenes* is a gram-positive environmental bacterium that survives as a saprophyte in soil but is capable of causing disease when ingested by susceptible individuals [1], [2], [3], [4], [5]. This facultative intracellular pathogen has been responsible for some of the most extensive and expensive food recalls in U.S. history, and invasive *L. monocytogenes* infections have resulted in thousands of reported illnesses and hundreds of deaths within recent years [3], [6], [7], [8], [9]. The transition from bacterial life in the outside environment to life within the mammalian cell cytosol requires the transcriptional upregulation of a number of gene products that contribute to host cell invasion, dissolution of the phagosomal membrane, replication within the cytosol, and bacterial spread to neighboring host cells [10], [11], [12], [13]. The expression of the gene products that enable *L. monocytogenes* to establish its intracellular replication niche is regulated by a transcriptional activator known as PrfA [12], [14], [15]. PrfA is essential for *L. monocytogenes* virulence as mutants lacking this regulator are unable to cause disease in mouse models of infection [16].

PrfA is a 27kDa protein that belongs to the cAMP receptor protein (Crp)-Fnr family of transcriptional regulators whose members appear to require the binding of a small-molecule cofactor for full activity [17], [18]. PrfA has been shown to be structurally similar to the most well studied member of this family, the *Escherichia coli* Crp protein [19]. PrfA monomers consist of an N-terminal β-barrel domain and a C-terminal DNA-binding helix-turn-helix (HTH) domain that are linked by a long α-helix that mediates monomer-monomer contacts and the formation of PrfA dimers [19]. The PrfA N-terminal domain consists of an eight-stranded antiparallel β-barrel; in Crp, this domain contains a conserved cyclic nucleotide monophosphate binding pocket that binds the Crp activating cofactor cAMP. Structural analysis of PrfA has indicated the presence of a putative cofactor binding pocket in a region similar to that of the Crp cAMP binding site, however in contrast to Crp, key residues involved in the binding of nucleotide sugars are absent in PrfA [19]. The absence of conserved residues that contribute to the binding of nucleotide sugars is consistent with studies reporting that neither cAMP nor cGMP serve as activating cofactors for PrfA [12], [18]. The nature of the cofactor that leads to PrfA activation is not presently known but has been speculated to be a host-derived small-molecule second messenger. In addition, functional linkages between carbon metabolism and PrfA-dependent virulence gene expression have led to the suggestion that available nutrients may serve to trigger the generation of the activating signal for bacteria located within host cells [20], [21], [22], [23], [24], [25].

In the absence of a known cofactor for PrfA, mutations within *prfA* have been identified (prfA* mutations) that serve to lock the protein in a constitutively active form [26], [27], [28], [29], [30], [31]. A number of prfA* mutations have been mapped to different regions within the protein, however the only structural information available thus far that describes the influence of a prfA* mutation on PrfA function relates to the prfA* G145S mutation located in the PrfA C-terminal domain [19]. The presence of the prfA* G145S mutation results in the repositioning of the PrfA helix-turn-helix DNA-binding domain so as to increase PrfA DNA-binding affinity. Other *prfA* mutations, including Y63C and Y154C, both of which map in or near the co-factor binding pocket region, appear to activate (Y63C) or inhibit activation (Y154C) of the protein without significant changes in PrfA DNA binding affinity; the mechanistic basis of action of these mutations remains unclear [26], [31]. While the prfA* Y63C and *prfA* Y154C mutations profoundly alter PrfA activity, they have provided few clues into the nature or identity of the PrfA activating cofactor.

In this study, we use electrostatic modeling to reveal that the putative cofactor binding pocket is characterized by a high density of positive charge resulting from the presence of three key lysine residues: K64 and K122 which are located at the opening of the pocket, and K130 which is buried deep within the pocket interior. Mutational analysis was used to investigate the contributions of these lysine residues to PrfA function and activation. Our data support a model in which the positive charge of the PrfA binding pocket plays a key role in cofactor binding and optimal PrfA activation within the cytosol of infected host cells.

## Results

### Electrostatic modeling reveals a high density of positive charge within the putative PrfA cofactor binding site

**Figure 1:**
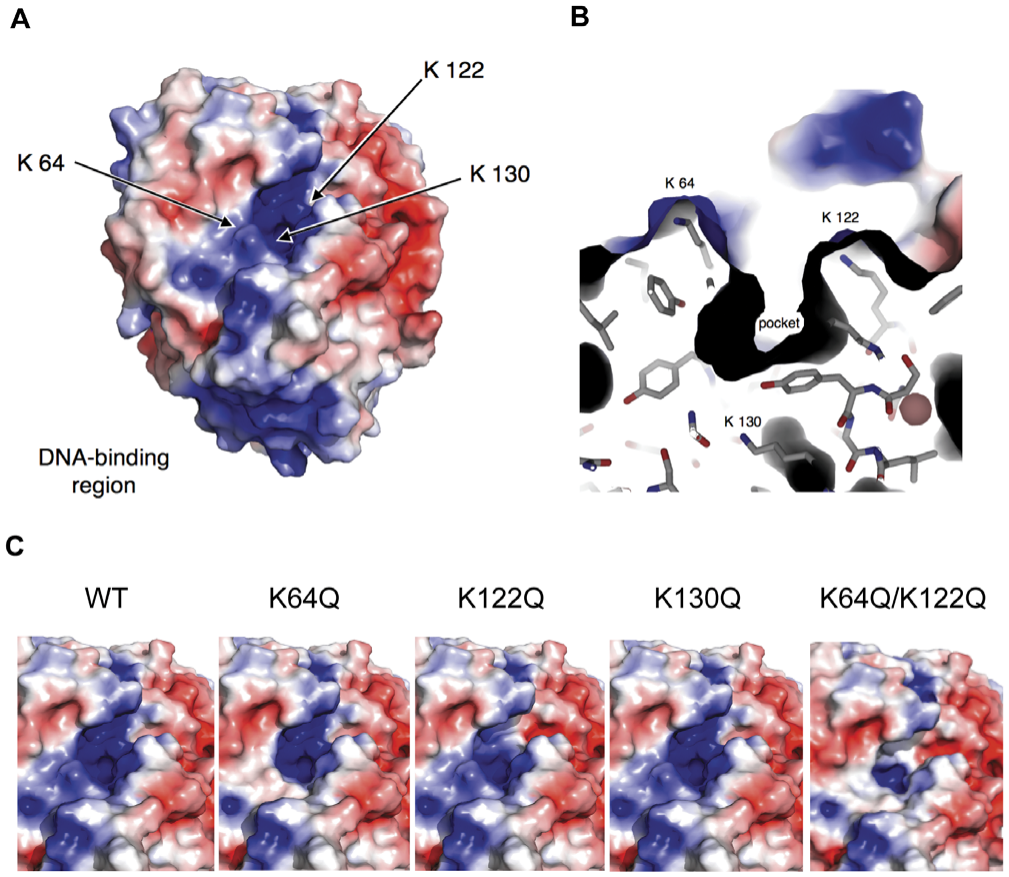
Electrostatic modeling of PrfA. **(A)** Electrostatic potential distribution on solvent-accessible surface of wild-type PrfA. Positive charge is indicated in blue, negative charge in red. Arrows point to lysine residues within the putative PrfA cofactor binding pocket. The positively charged DNA binding region located at the bottom of the PrfA monomer is indicated. **(B)** Close-up of lysine residues and pocket for wild-type PrfA, in approximately the same orientation as **A**. **(C)** Electrostatic surface potentials at putative cofactor binding pocket for wild-type PrfA and lysine mutants. The electrostatic potentials range from -4kT/e (red) to +4 kT/e (blue).

PrfA is a 237 amino acid protein with an N-terminal domain consisting of residues 1–108 and a C-terminal comprised of residues 138–237 [19]. The N-terminal domain is comprised of eight-stranded antiparallel β-barrel sheets flanked by two α-helices (αA and αB), while the C-terminal is made up of six α-helices and four antiparallet β-barrel sheets, two of which (αE and αF) comprise the helix-turn-helix DNA binding motif. The N and C terminal domains are linked via a long alpha helix (αC) consisting of residues 109–137 [19]. The predicted cofactor binding site identified by Eiting *et al* [19] in the PrfA x-ray crystal structure consists of a tunnel-like region located between the N-terminal β-barrel and C-terminal DNA-binding domains within the protein monomer (Fig. 1A–B). Electrostatic modeling of PrfA was used to probe the physical nature of the putative binding pocket and this approach revealed a high density of positive charge stemming from the presence of three lysine residues: K64, K122, and K130 (Fig. 1A). K64 and K122 are located on opposite sides of the opening of the pocket (Fig. 1A–B), while the K130 lysine is buried deep within the pocket interior. K64 is located within beta sheet β5 (residues 56–64), while K122 and K130 are located within helix αC (residues 109–137) [19]. In the structurally related activator Crp, β4–β5 fold over the cAMP-binding pocket such that the binding of cAMP bends the αC helix and results in a structural shift that repositions the DNA binding HTH motif to enhance DNA-binding [19]. A similar repositioning of the DNA HTH domain has been shown to occur in PrfA as a result of a G145S substitution; this mutation enhances PrfA DNA binding affinity and results in the constitutive activation of PrfA in the absence of cofactor [19], [27].

### Substitution of the lysine residues at position 64, 122, or 130 does not impact PrfA protein stability or dimer formation

The high density of positive charge revealed via electrostatic modeling of the putative PrfA cofactor binding pocket suggested that charge may play a role in PrfA cofactor binding. To probe the contributions of pocket charge to PrfA function and to cofactor-induced activation, mutant strains containing the substitution of glutamine for the positively charged lysine residues were constructed via complementation of *L. monocytogenes* Δ*prfA* strains with the plasmid single copy integration vector pPL2 containing the mutant *prfA* alleles (along with all relevant promoters that contribute to *prfA* expression [30]), and compared to *L. monocytogenes* Δ*prfA* strains containing pPL2 encoding the wild type *prfA* allele. PrfA proteins containing the substitution of glutamine for lysine at position 64 (K64Q) or at position 122 (K122Q) would be predicted to exhibit a limited decrease in overall positive charge at the opening of the pocket, while the simultaneous substitution of both lysines would result in a more substantial reduction in positive charge (Fig. 1C). The lysine residue at position 130 is buried deep within the interior of the pocket, and the substitution of this lysine for glutamine would be anticipated to significantly influence PrfA structure while not visibly affecting the positive charge at the pocket entrance (Fig. 1C). If K64, K122, and/or K130 contribute to the binding of the PrfA cofactor via positive charge, neutral substitutions at these lysine residues would be anticipated to interfere with cofactor binding and PrfA activation.

**Figure 2:**
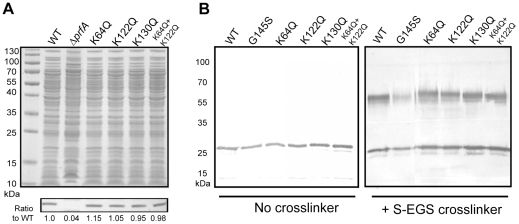
Substitution of *prfA* lysine residues for glutamines does not affect PrfA stability or the ability to form homodimers. **(A)** Western blot of PrfA protein. Top panel: 20 µl of isolated bacterial cytoplasmic fractions normalized based on cell density were loaded onto a 12% Bis-Tris polyacrylamide gel and proteins were separated by gel elecrophoresis. Polypeptides were visualized by staining with Coomassie blue. Bottom panel: PrfA protein was detected in the cytoplasmic fractions of the various *L. monocytogenes* strains using a monoclonal antibody directed against PrfA. All *prfA* lysine substitution mutants synthesized similar amounts of PrfA protein in comparison to the wild-type strain. Numbers below Western panel indicate relative amounts of protein as determined by densitometry using ImageJ software (http://rsbweb.nih.gov/ij/download.html) in comparison to wild type PrfA. **(B)** Chemical crosslinking of purified PrfA. 500 ng of purified PrfA proteins were chemically crosslinked with 10 µM of sulfo-ethylene glycol bis[succinimidylsuccinate] (S-EGS) for 1 hour at room-temperature followed by SDS-PAGE and western blotting for detection of PrfA dimers. All PrfA lysine mutant proteins formed homodimers at levels similar to those observed with the wild-type protein. All data is representative of at least three independent experiments.

Western blot analysis was used to confirm the expression and stability of PrfA as encoded by the *prfA* lysine substitution mutant strains (Fig. 2A). The analysis of cytoplasmic fractions of bacterial lysates prepared from cells grown to mid-exponential phase indicated that strains containing *prfA* K64Q, K122Q, K130Q and K64Q/K122Q mutations resembled wild type strains in terms of PrfA synthesis and stability (Fig. 2A). As PrfA forms dimers in solution as detected both by x-ray crystallography structural analysis and by chemical cross-linking studies [18], [19], [31], [32], mutant dimer formation was examined using chemical cross-linking agents (Fig. 2B). Purified wild type PrfA, PrfA* (G145S), and K64Q, K122Q, K130Q, and K64Q/K122Q were incubated with the chemical crosslinkers sulfo-ethylene glycol bis[succinimidylsuccinate] (S-EGS), which has a 16 Ångstrom linker arm, and *Bis* [sulfosuccinimidyl] suberate (BS^3^), which has an 11 Ångstrom linker arm; both of these reagents react with free amine groups. All of the PrfA lysine substitution mutants formed homodimers as well as the wild-type protein, while PrfA G145S exhibited reduced dimer formation as previously reported [31] (Fig. 2C and data not shown). These data confirm that the K64Q, K122Q, and K130Q lysine substitution mutant proteins are stably expressed in *L. monocytogenes* and retain sufficient structural conformation for dimer formation.

### Variable impact of the PrfA lysine substitutions on *in vitro* PrfA-dependent gene expression

**Figure 3:**
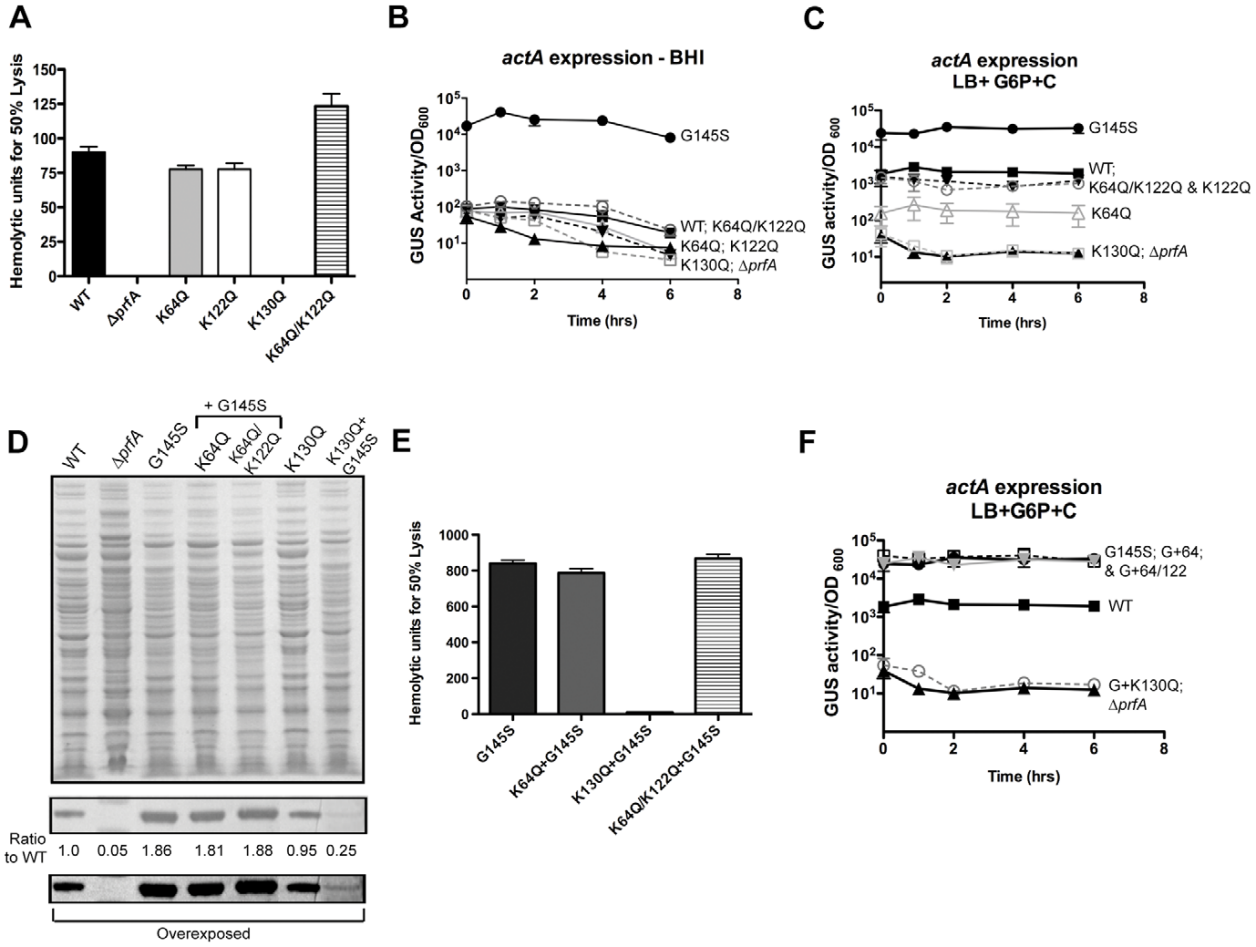
Effects of *prfA* lysine substitutions on PrfA-dependent virulence gene expression. **(A)** Assessment of LLO-associated hemolytic activity by measuring lysis of sheep erythrocytes from serial dilutions of bacterial culture supernatants of strains grown for 5 hours at 37°C with shaking in LB broth. Hemolytic activity was determined as the reciprocal of the supernatant dilution at which 50% lysis of erythrocytes was observed. Data shown represents the mean ± SEM activity measured in triplicate for three independent experiments. **(B)** Measurement of the levels of PrfA activity in broth culture as assessed by *actA* expression. PrfA-dependent virulence gene expression was measured by monitoring the levels of GUS activity of the various *L. monocytogenes* strains containing *actA-gus* transcriptional reporter fusions. Bacterial strains were grown in BHI at 37°C with shaking and GUS activity was assayed from normalized samples collected at the indicated time points. Each data point is the mean ±SEM GUS activity measured in duplicate, and the data shown is representative of at least two independent experiments. **(C)** Measurement of the levels of PrfA activity in broth culture as assessed by *actA* expression under *in vitro* inducing conditions. Assays were carried out as described in (B) except under inducing conditions in which bacterial strains were grown in LB broth containing 0.2% activated charcoal (C) and 25 mM glucose-6-phosphate (G6P). Data is representative of at least three independent experiments. **(D)** Western blot of PrfA protein from cytoplasmic fractions of *L. monocytogenes prfA* lysine mutants containing a prfA* G145S mutation. Numbers reflect the relative amounts of mutant PrfA protein in comparison to wild type protein levels as determined by densitometry using ImageJ software. Bottom panel represents a prolonged exposure to aid in the detection of the PrfA K130Q G145S protein band. Data is representative of at least three independent experiments. **(E)** Assessment of LLO-associated hemolytic activity as described above in panel A in the presence of the prfA* G145S allele. **(F)** Measurement of the levels of PrfA activity in the presence of the prfA* G145S mutations as determined by *actA* expression (as described above in panel C). For both (E) and (F) panels, data is representative of at least three independent experiments.

PrfA is required for the expression of the majority of *L. monocytogenes* gene products associated with intracellular survival and virulence within mammalian hosts [1], [11], [12], [13], [14]. While the expression of PrfA-dependent genes is induced within infected host cells, the activity of a number of gene products can still be detected at low levels during bacterial growth in broth culture [26], [33], [34], [35]. We therefore first assessed the influence of the PrfA lysine substitutions on the expression of selected *L. monocytogenes* virulence gene products *in vitro*. The secreted hemolysin LLO is produced at low levels by bacteria grown in broth culture and contributes to bacterial lysis of the phagosome following host cell entry [34], [36]. Secreted LLO-associated hemolytic activity can be assessed by monitoring the lysis of sheep red blood cells in the presence of bacterial supernatant fractions. *L. monocytogenes* strains containing the *prfA* K64Q and K122Q mutations secreted levels of LLO-associated hemolytic activity that were comparable to wild-type *L. monocytogenes*, whereas the *prfA* K130Q mutant produced no detectable LLO activity (Fig. 3A). Interestingly, the combined K64Q/K122Q mutant exhibited slightly increased amounts of LLO-associated hemolytic activity compared to the wild type strain.

The expression of the PrfA-dependent gene product encoded by *actA* was measured using strains containing an *actA-gus* transcriptional reporter gene fusion within the *L. monocytogenes* chromosome, for which *ß*-glucuronidase (GUS) activity serves as a read-out for *actA* expression. Wild type *L. monocytogenes* and mutant strains grown in BHI at 37°C were assayed for GUS activity at varying time points of growth. The constitutively active prfA* G145S mutant was included to provide a comparison for the expression of high levels of *actA* expression as reflected by GUS activity. All strains containing *prfA* K64Q, K122Q, K130Q, or K64Q/K122Q mutation were found to express very low levels of *actA* similar to those expressed by wild-type *L. monocytogenes* in broth culture (conditions under which PrfA is not activated), and all were significantly lower than those expressed by the prfA* G145S strain (Fig. 3B). These data combined with the LLO-associated hemolytic data indicate that the *prfA* K64Q and K122Q mutations do not appear to significantly impact low level *in vitro* PrfA-dependent gene expression. In contrast, the deep pocket K130Q substitution mutant eliminated PrfA-dependent *in vitro* expression of LLO.

In the absence of host cell induction of PrfA-dependent gene expression, it has been observed that PrfA activity can be increased by the inclusion of charcoal and glucose-6-phosphate in bacterial growth media [33], [37]. To assess the ability of the various *L. monocytogenes prfA* mutant strains to induce gene expression under *in vitro* inducing conditions, bacterial strains were grown in LB broth plus 25 mM glucose-6-phosphate and 0.2% activated charcoal and levels of *actA* expression were determined. *actA* expression was observed to increase approximately 40-fold for strains containing wild type *prfA* in comparison to strains grown in BHI (Fig. 3C). *actA* expression was also increased for strains containing the *prfA* K64Q, K122, and K64Q/K122Q mutants in comparison to growth in BHI, however the induction of *actA* expression observed was significantly less for *prfA* K64Q strains (4-fold induction vs 40-fold induction for wild type *prfA*) (Fig. 3C). The *in vitro* induction of *actA* expression was also reduced in *prfA* K122Q and K64Q/K122Q strains, however the difference in the levels of *actA* expression observed between inducing conditions and BHI was less significant in comparison to wild type *prfA* strains (24-fold and 21-fold induction, respectively).

The use of *in vitro* conditions to activate PrfA-dependent gene expression is necessary for the detection of secreted PlcB phospholipase activity on egg yolk agar plates [37]. Growth of bacteria on LB agar plates containing 5% egg yolk, 25 mM of glucose-6-phosphate, and 0.2% activated charcoal resulted in the production of a defined zone of secreted phospholipase activity surrounding the wild type strain (Fig. S1). Consistent with the *actA* expression data shown in Fig. 3C, strains containing the mutationally activated *prfA* G145S allele exhibited larger zones of activity, while the *prfA* lysine substitutions mutants produced reduced levels of secreted phospholipase (*prfA* K64Q, K122Q, K64Q/K122Q) or no detectable activity (*prfA* K130Q) in comparison to wild type (Fig. S1). The reduced induction of *actA* expression and PlcB-dependent phospholipase activity by the *prfA* K64Q and K122Q strains in the presence of glucose-6-phosphate and activated charcoal suggests that these *prfA* mutations impair the ability of the protein to become fully activated under *in vitro* inducing conditions. The lack of any detectable activity associated with the K130Q mutation suggests that this substitution functionally inactivates the protein.

Given the apparent defects in the ability of the *prfA* lysine substitution mutants to increase PrfA-dependent gene expression under *in vitro* inducing conditions, we investigated whether the mutant proteins could become functionally activated via the introduction of the prfA* G145S mutation. As mentioned previously, the *prfA* G145S substitution leads to the repositioning of the HTH DNA binding domain and enables PrfA activation in the absence of cofactor binding [18], [19]. The introduction of the prfA* G145S mutation into the *prfA* K64Q or the *prfA* K64Q/K122Q mutant resulted in the expression of stable protein as detected by Western blot analysis, with amounts produced that were comparable to the levels observed for strains containing the prfA* G145S allele alone (Fig. 3D). *prfA* G145S, *prfA* G145S K64Q, and *prfA* G145S K64Q/K122Q strains produced approximately twice as much PrfA as did strains containing wild type *prfA*, suggesting that the prfA* mutation was dominant over these mutations and stimulated additional *prfA* expression from the upstream PrfA-dependent *plcA* promoter [38], [39]. Interestingly, although a K130Q substitution did not dramatically affect PrfA protein synthesis or stability, the addition of the prfA* G145S mutation to K130Q reduced PrfA protein levels by more than 4-fold (Fig. 3D). Based on the position of the K130 residue within the interior of putative PrfA cofactor binding pocket combined with the lack of detectable PrfA activity associated with the *prfA* K130Q mutant strains, it would appear that the *prfA* K130Q G145S double substitution elicits structural changes that reduce PrfA protein stability.

The introduction of the prfA* G145S mutation in combination with the *prfA* K64Q or K122Q mutations resulted in levels of *actA* expression, secreted hemolytic activity, and phospholipase activity that were essentially identical to strains containing prfA* G145S alone, indicating that the activation mutation was fully dominant (Fig. 3E, F, and Fig. S1). In contrast, combining the prfA* G145S mutation with *prfA* K130Q did not result in any measurable increase in PrfA-dependent gene expression, a result consistent with the dramatic reduction in the stability of this protein in *L. monocytogenes* (Fig. 3D). Therefore, in contrast to the *prfA* K130Q G145S mutant, PrfA protein containing the K64Q and K122Q substitutions retain the ability to become mutationally activated by the G145S mutation, suggesting that these mutations influence the putative cofactor binding pocket without preventing PrfA conformational changes associated with activation.

### The substitution of PrfA lysine residues negatively impacts PrfA binding of DNA

**Figure 4:**
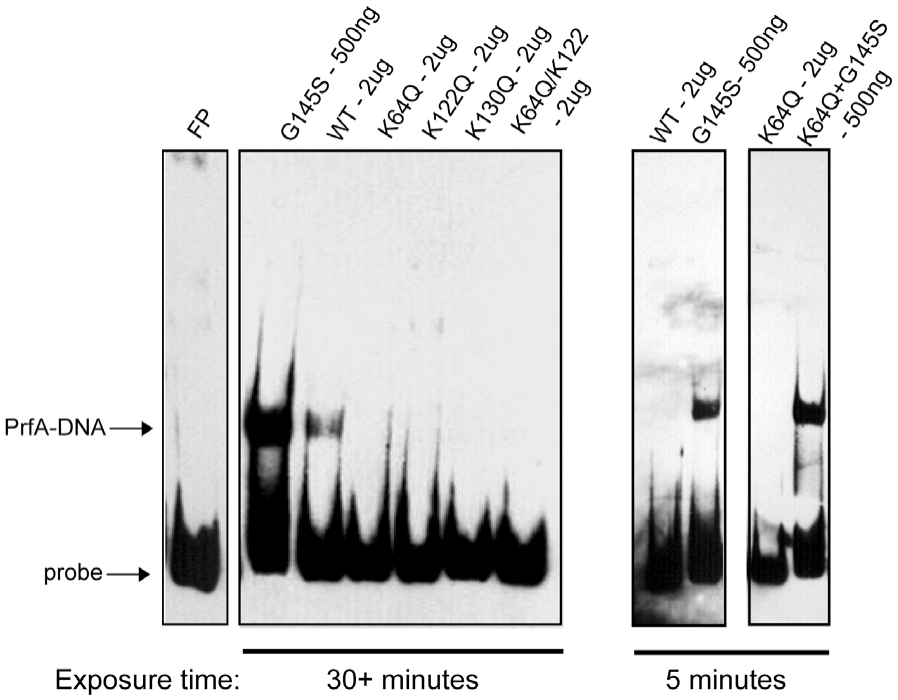
Substitution of the *prfA* lysine residues affects formation of DNA-protein complexes. The binding ability of the various PrfA lysine mutant proteins to a biotin-labeled *hly*-promoter DNA fragment was assessed by electrophorectic mobility shift assays (EMSAs). The *prfA* K64Q, K122Q, K130Q, and K64Q/K122Q mutants all exhibited defects in their ability to form DNA-protein complexes. PrfA lysine mutant complexes were not detectable even with the addition of increased protein or by exposing the film for a longer duration of time (data not shown). The ability to form complexes was restored in the presence of the PrfA* G145S mutation (shown for K64Q) to the levels observed with PrfA* G145S. PrfA - DNA-protein complexes are indicated. FP  =  free probe, no protein added. Data shown is representative of at least three independent experiments.

The putative PrfA cofactor binding pocket is located within the N-terminal domain of PrfA, somewhat removed from the C-terminal DNA binding region (Fig. 1A). Based on structural and functional analogies with Crp, PrfA cofactor binding is anticipated to trigger a conformational change in the protein that repositions the DNA binding region and increases DNA binding affinity. Alterations within the pocket, such as amino acid substitutions, might therefore be anticipated to potentially influence PrfA DNA binding via changes in C-terminal protein conformation distally triggered by the N terminal domain. We examined the DNA binding capacity of the purified mutant proteins using electromobility shift assays (EMSAs) in conjunction with the PrfA-dependent *hly* promoter. Previous work has indicated that PrfA* mutants exhibit high affinity site-specific DNA binding in the absence of any cofactor, while wild type protein exhibits only weak but specific binding even with high concentrations of purified PrfA protein [18], [29], [31], [32]. As previously observed, purified wild-type PrfA bound DNA with a low affinity, resulting in a partial shift of the labeled DNA probe, whereas PrfA G145S bound DNA with high affinity, resulting in a substantially larger proportion of PrfA bound DNA even in the presence of lower concentrations of protein (Fig. 4). All of the PrfA lysine substitution mutant proteins tested were unable to form detectable PrfA-DNA complexes in comparison to wild type PrfA (Fig. 4), even in the presence of increased amounts of purified protein (data not shown). Structural changes within the cofactor binding pocket can therefore negatively impact PrfA DNA binding.

### Conformational changes induced by PrfA lysine substitutions can be detected by limited proteolytic digestion

**Figure 5:**
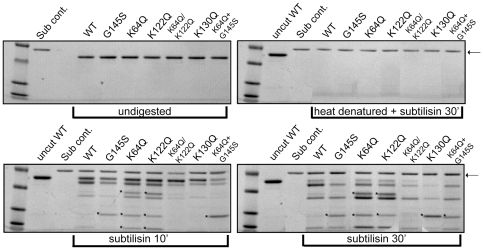
Comparison of protein conformation by limited proteolytic digestion. 2 µg of wild type, PrfA* G145S and the various PrfA lysine mutant proteins were digested with 500 ng of subtilisin for 10 and 30 minutes at room-temperature. 1 µM of PMSF was added and protein fragments were separated by SDS-PAGE and visualized by Coomassie staining. Untreated protein samples (uppermost left panel) and also heat-denatured samples treated with subtilisin (uppermost right panel) were included as controls to demonstrate that denatured samples were equally susceptible to enzymatic digestion. Arrow indicates the position of subtilisin and asterisks (*) denote fragments that were not observed for the wild type protein. Gels are representative of three independent experiments.

Given that *prfA* lysine K64Q, K122Q, K130Q, and K64Q/K122Q mutations result in observable alterations in PrfA stability and/or *in vitro* activity, we assessed and compared wild type and mutant PrfA protein conformation using limited proteolytic digestion of purified protein. This simple, valid, and established approach has been used to examine structural changes in Crp and can be used to visualize conformational changes in PrfA, including those that occur as a result of PrfA activation [31], [40], [41]. Purified PrfA wild type and mutant proteins containing each of the lysine substitutions as well as the double PrfA K64Q G145S substitution were subjected to limited digestion with the protease subtilisin. The PrfA K130Q G145S protein was found to be unstable and was therefore not included in the analysis. Polypeptide fragments resulting from limited protease digestion were separated by SDS-polyacrylamide gel electrophoresis and visualized by Coomassie staining (Fig. 5). Heat denatured PrfA samples were additionally subjected to subtilisin digestion to confirm that all protein samples were equally susceptible to proteolytic cleavage when denatured. As anticipated, all protein samples were completely digested following heat denaturation with only a band representing subtilisin remaining (Fig. 5, top right panel). As previously reported, conformational changes in PrfA protein structure resulting from the prfA* G145S mutation could be distinguished in the mutant versus the wild type protein based on the mutant's increased susceptibility to proteolysis and altered protease digestion pattern (Fig. 5, lower panels). The PrfA K64Q G145S mutant exhibited a digestion pattern that was similar to that of PrfA G145S, consistent with the apparent functional dominance of the prfA* mutation over that of K64Q. Interestingly, both the PrfA K64Q and the K122Q substitution mutant proteins exhibited slightly different subtilisin digestion patterns from those observed for the wild type protein or the K64Q/K122Q mutant (which somewhat more closely resembled wild type PrfA) (Fig. 5). Surprisingly, while still slightly distinct, the digestion pattern of the PrfA K130Q mutant resembled that of the wild type protein despite the lack of activity associated with *prfA* K130Q mutation in *in vitro* assays (Fig. 5); this result suggests that the impact of K130Q on PrfA function is not associated with gross alterations in PrfA conformation [a result also consistent with PrfA K130Q dimer formation (Fig. 2)].

### *L. monocytogenes* strains containing the *prfA* K64Q and K122Q mutations are modestly impaired for intracellular growth and cell-to-cell spread

**Figure 6:**
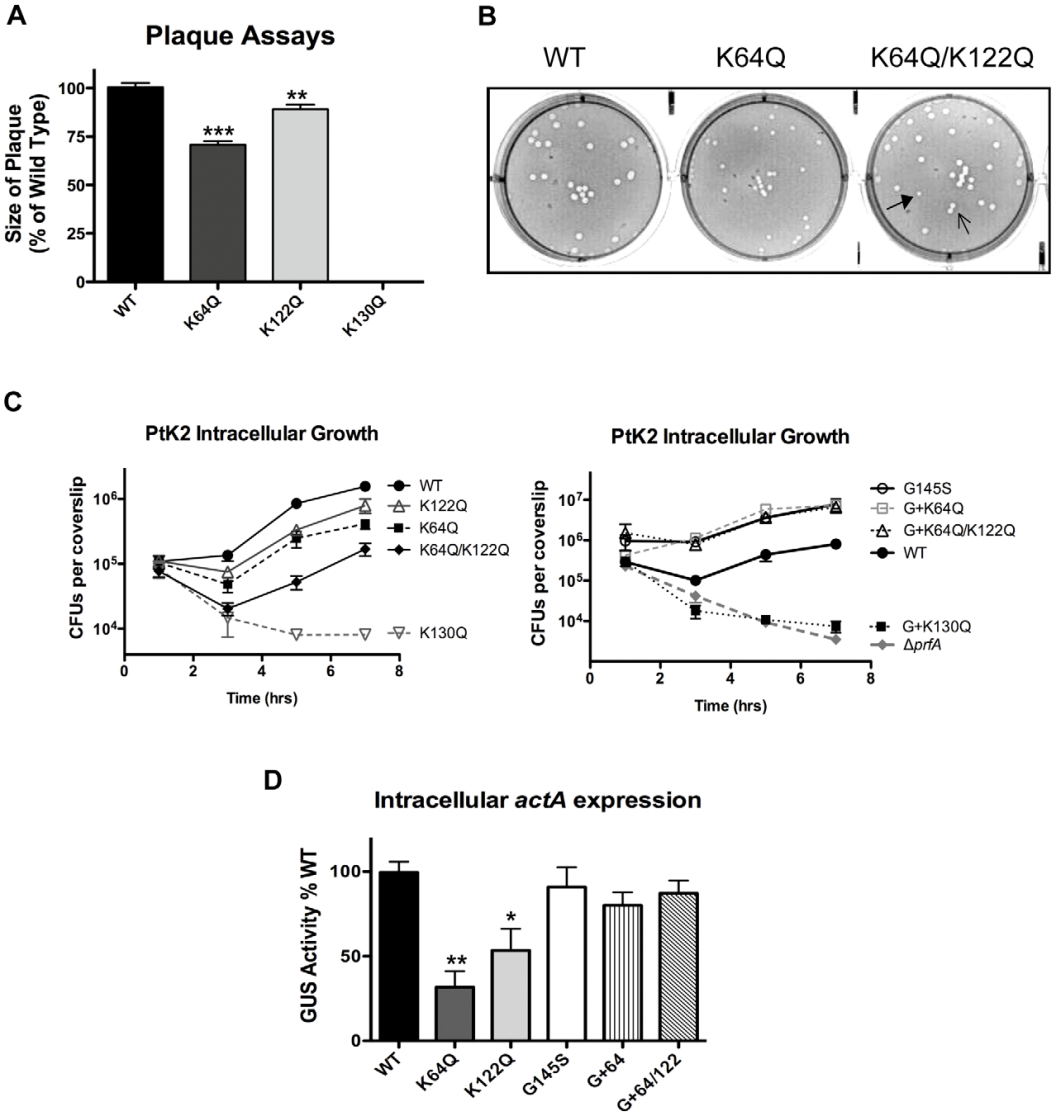
The *prfA* K64Q and K122Q lysine substitution mutants are impaired for intracellular PrfA activation, while the K130Q mutant is defective for vacuole escape. **(A)** The ability of the specified *prfA* lysine mutants to grow intracellularly and spread from cell-to-cell was determined by assessing plaque formation in murine L2 fibroblasts. Monolayer of L2 cells were infected with the bacterial strains at an MOI of 10∶1. Gentamicin was added after 1 hour, and plaques were visualized three days post-infection. Data shown is representative of three independent experiments done in duplicate. **(B)** Measurement of plaque formation of the K64Q/K122Q mutant. Solid arrow head indicates a small plaque and the line arrow indicates an intermediate plaque size. **(C)** Intracellular growth of *prfA* mutants strains in PtK2 epithelial cells. Bacterial intracellular growth was measured by infecting monolayers of PtK2 cells grown on glass coverslips (cs) with the indicated strains at an MOI of 100∶1. Gentamicin was added 1 hour post-infection (p.i.), cells were washed, and cs were removed at the indicated time points. Host cells were lysed and the amount of intracellular bacteria per cs was enumerated. Left panel: *prfA* lysine mutants. Right panel: *prfA* lysine mutations combined with the prfA* G145S allele. Data shown represents the mean +SEM of three independent experiments done in triplicate. **(D)** Intracellular *actA* expression. Intracellular *actA* expression was determined by measuring GUS activity from lysed PtK2 cells infected with the indicated *prfA* lysine mutants at an MOI of 100∶1 at 5 hours post-infection. The number of CFU per dish was determined following the lysis of PtK2 cells grown on coverslips in duplicate dishes and GUS activity per dish was calculated as described by Moors *et al* [57]. Each assay was done in duplicate and data shown represents the mean ±SEM and is representative of three independent experiments. Statistical analysis for panels A and D were done using an unpaired two-tailed student *t*-test (GraphPad Prism V.5.0A) where ***p≤0.0005, **p≤0.005, and *p≤0.05.

The data obtained thus far suggested that the PrfA lysine substitution mutations functionally impact PrfA activity and reduce the ability of PrfA to become activated outside of host cells. PrfA appears to become fully activated as *L. monocytogenes* gains access to the host cell cytosol, resulting in the full induction of PrfA-dependent gene products that contribute to intracellular bacterial growth and cell-to-cell spread [35]. To assess the influence of the PrfA cofactor binding site mutations on *L. monocytogenes* bacterial invasion, intracellular growth, and cell-to-cell spread within host cell monolayers, strains containing the *prfA* lysine substitutions were analyzed for the ability to form zones of clearing or plaques in monolayers of mouse fibroblast cells [42]. Strains containing *prfA* K130Q failed to form detectable plaques in fibroblast monolayers, a result consistent with this mutant's lack of detectable LLO activity and failure to mediate phagosome lysis (Fig. 6A). Strains containing the *prfA* K64Q, *prfA* K122Q, or the *prfA* K64Q/K122Q mutation formed plaques with approximately the same frequency as the wild-type strain, indicating that these mutations do not reduce host cell invasion for fibroblast cells (Fig. 6B). However, the sizes of the plaques formed by the *prfA* K64Q mutant were significantly smaller than those formed by wild-type *L. monocytogenes* (60–70% of the wild type size), and modest reductions in plaque size were also observed for cells infected with the *prfA* K122Q mutant (80–90% of the wild-type size). Similar reductions in plaque size have been previously associated with bacterial defects in cell-to-cell spread [43], [44]. Interestingly, strains containing the *prfA* K64Q/K122Q mutation produced plaques of varying sizes, ranging from 60%–100% of the size of wild type plaques, with most (>75%) similar in size to those produced by strains containing wild type *prfA* or the *prfA* K122Q mutant (Fig. 6B). When individual bacterial colonies of *prfA* K64Q K122Q bacteria were isolated from either small or large plaques and re-tested for plaque formation, all isolates continued to form plaques of mixed sizes (data not shown). This result is suggestive of a stochastic event, in which some bacteria within a population exhibit reduced efficiency of cell-to-cell spread perhaps as a result of delayed induction of *actA* expression, whereas others replicate and move with wild type kinetics.

Bacterial uptake and replication were further assessed for growth in PtK2 epithelial cell lines. The *prfA* K64Q, K122Q, and K64Q/K122Q mutants were capable of intracellular replication although bacterial invasion appeared somewhat reduced (indicated by the reduction in the numbers of bacteria protected from gentamicin at one hour post-infection) (Fig. 6C, left panel). Microscopic examination of cells infected with the *prfA* K64/K122Q mutant revealed a pleiotropic pattern of intracellular growth, with evidence of efficient intracellular replication and cell-to-cell spread in some populations of infected cells, and reduced spread in others (data not shown), consistent with the mixed plaque phenotype observed for this mutant. The *prfA* K130Q mutant was completely defective for intracellular replication (Fig. 6B), and failed to associate with host actin (data not shown), consistent with a failure of this mutant to mediate LLO-dependent escape from the phagosome.

The prfA* G145S mutation exhibited a dominant phenotype when combined with either the single *prfA* K64Q or double *prfA* K64/K122Q substitutions; mutant bacteria containing *prfA* G145S were hyper-invasive and formed increased numbers of plaques that were similar in size to those formed by wild type *L. monocytogenes* (Fig. 6C right panel and data not shown).

### Strains containing *prfA* K64Q and K122Q mutations exhibit reduced induction of *actA* expression within the cytosol of infected host cells

The expression of the *actA* gene product (required for bacterial actin-based motility) is completely dependent upon PrfA and, following the activation of PrfA within the cytosol of infected host cells, *actA* expression increases several hundred fold in comparison to expression levels observed in broth culture [26], [35], [45], [46]. Given that the PrfA cofactor binding site mutants appeared defective for cell-to-cell spread based on their reduced plaque size in tissue culture cell monolayers, we examined intracellular *actA* induction in PtK2 epithelial cells. Strains containing the *prfA* K64Q and K122Q mutations exhibited an approximate 3-fold reduction in the levels of intracellular *actA* expression in comparison to the wild-type strains (Fig. 6D). Although somewhat modest, this reduction in *actA* expression could account for the reduced efficiency of cell-to-cell spread exhibited by the *prfA* K64Q and K122Q mutant strains as it has been demonstrated that a threshold level of ActA must accumulate before actin-based motility begins [44]. A 3-fold reduction in *actA* transcription would therefore be anticipated to lengthen the amount of time spent by *L. monocytogenes* synthesizing ActA within the cytosol before bacterial movement could be initiated. *prfA* G145S, and the K64Q or K64Q/K122Q mutants in the presence of the *prfA* G145S allele all exhibited similar levels of intracellular *actA* induction as the wild type strain (Fig. 6D), a result which indicates that these mutants are activated to maximal levels within the cytosol.

### *prfA* lysine mutants are attenuated for virulence in murine infection models

**Figure 7:**
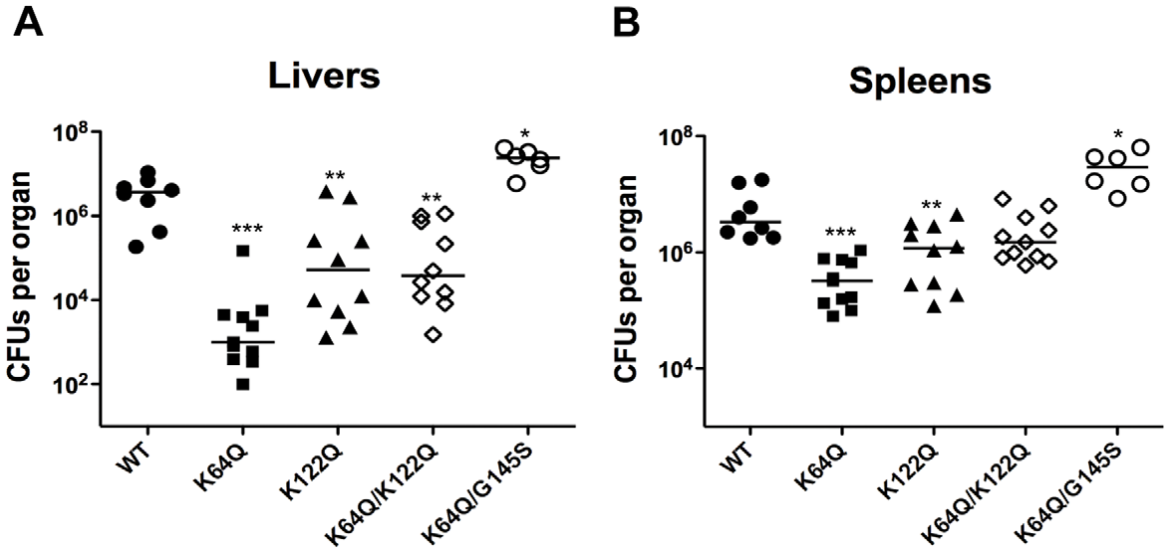
PrfA lysine residues contribute to virulence of *L. monocytogenes.* Swiss Webster mice were intravenously infected with 2×10^4^ CFU through the tail vein. At 48 hours post-infection, the livers (A) and spleens (B) were harvested, homogenized, and plated for bacterial CFUs. Each datum point represents one mouse, and the solid lines denote the median for each data group. Data was obtained from two independent experiments. Asterisks indicate statistical significance of *p≤0.05, **p≤0.005, and ***p≤0.0005 using an unpaired two-tailed student *t*-test (GraphPad Prism V.5.0A).

Based on data presented thus far, the *prfA* K64Q and K122Q mutations appeared to interfere with the ability of PrfA to become fully activated in host cells, whereas the K130Q mutation completely abolished PrfA activity. To further investigate the contributions of the PrfA K64 and K122 residues to PrfA activity *in vivo*, mice were intravenously infected with the single and double mutant strains and bacterial burdens were determined for liver and spleen at two days post-infection. All of the strains containing *prfA* K64Q or K1221Q mutations were significantly attenuated for virulence in mice (Fig. 7). The *prfA* K64Q mutant was the most severely attenuated, with bacterial numbers recovered from the liver and spleen that were approximately 250-fold lower and 10-fold lower, respectively, in comparison to mice infected with wild type bacteria (Fig. 7). The bacterial burdens recovered from the livers and spleens of mice infected with the *prfA* K122Q mutant were 10-fold and 5-fold lower, respectively, in comparison to wild type infected mice. Interestingly, while the *prfA* K64Q/K122Q mutant was attenuated for virulence based on the numbers of bacteria recovered from the liver, no significant difference was observed between the mutant and wild type bacteria with respect to bacterial numbers recovered from the spleen (Fig. 7). Thus, instead of the *prfA* K64Q/K122Q mutant exhibiting levels of attenuation comparable or more severe than those exhibited by the *prfA* K64Q single mutation strain, the double substitution mutant was instead similar to or slightly more virulent than the *prfA* K122Q mutant. The introduction of the prfA* G145S mutation fully restored virulence to the severely attenuated *prfA* K64Q strain, demonstrating once again that mutational activation of PrfA compensated for the binding site mutation defect.

## Discussion

As the primary regulator of bacterial virulence gene expression, PrfA plays a pivotal role in enabling *L. monocytogenes* to transition between the lifestyle of an environmental organism and that of an intracellular pathogen replicating within the cytosol [1], [10], [47]. The appropriate modulation of PrfA activity is critical for *L. monocytogenes* fitness. Constitutive PrfA activation reduces bacterial fitness outside of host cells, however an inability to fully activate PrfA in the cytosol reduces bacterial fitness within the host [20]. While significant progress has been made towards defining the physiological consequences of PrfA activation with respect to virulence factor secretion and bacterial survival, the signal that triggers PrfA activation remains unknown. PrfA activation is believed to occur via the binding of a small cofactor molecule within a structural pocket located in the N terminus of the protein [19]. In this study, we have identified a high density of positive charge associated with the putative PrfA cofactor binding pocket and explored the functional consequences of altering pocket charge via amino acid substitutions. Our results indicate that the positive charge of the cofactor binding pocket contributes to the process of PrfA activation, presumably by enhancing or stabilizing cofactor binding.

The PrfA K64 residue located within the N-terminal β5 sheet and the K122 residue located within the extended αC helix are in close proximity to one another and are located at the opening of the binding pocket (Fig. 1). The accessibility of these two lysines suggests that they could contribute to the initial binding or recruitment of the PrfA activating ligand. The substitution of either the K64 or the K122 residue with glutamine resulted in similar but distinguishable phenotypes as both were compromised for full PrfA activation. The K64Q substitution mutant exhibited the most significant defect in intracellular spread (Fig. 6) and was most severely attenuated for virulence in murine infection models (Fig. 7). The *prfA* K122Q mutant exhibited more modest defects in the induction of intracellular *actA* expression and cell-to-cell spread (Fig. 6), suggesting that its contributions to PrfA activation may be less important than those of the K64 residue. Interestingly and somewhat surprisingly, the combined *prfA* K64Q/K122Q double substitution mutant did not appear to severely impact PrfA activity even though the substitution of both lysine residues is predicted to have a dramatic effect on neutralization of the charge of the pocket (Fig. 1). The double substitution mutant did not exacerbate the effects observed for the single substitution mutants, but rather appeared to restore a portion of activity in comparison to *prfA* K64Q single substitution strains. It is possible that the charge neutralization of both lysine residues may potentially mimic charge neutralization that occurs as a result of cofactor binding. The charge neutralization of both residues restored a limited degree of PrfA function and activity *in vitro,* but may also have inhibited full PrfA activation by reducing the affinity of cofactor binding. Full PrfA activation was restored to both the *prfA* K64Q or K64Q/K122Q mutants by the introduction of the prfA* G145S mutation, suggesting that prfA* G145S-induced conformational changes were dominant over any potential alterations in cofactor binding.

The PrfA K130 residue is also located within αC helix but, unlike residues K64 and K122, is buried deep within the cavity of the pocket (Fig. 1B). The substitution of this residue with a glutamine virtually eliminated all detectable PrfA-dependent activity without grossly altering the PrfA protein confirmation as detected by limited protease digestion (Fig. 5), or affecting its ability to form homodimers (Fig. 2). The change in conformation imposed by the K130Q mutation was, however, of sufficient magnitude so as to prevent PrfA activation via the prfA* G145S mutation. Structural analysis of the PrfA* G145S mutant has indicated that a new hydrogen bond is formed between K130 and Q146 as a result of the G145S mutation [19]. K130 and Q146 may similarly interact as result of the conformational changes induced by cofactor binding, and the substitution of the lysine 130 to glutamine would be anticipated to eliminate this interaction and may thus impact PrfA activation. It is unusual to have a positively charged amino acid such as lysine buried so deeply within the protein interior; the location of K130 combined with the phenotype of the substitution mutant indicates a critical contribution of this residue to PrfA structure and function.All of the PrfA lysine substitution mutant proteins exhibited defects in DNA binding as indicated by gel mobility shift assays. This result suggests that alterations within the N terminal pocket can influence DNA binding by the C terminal domain of the protein. This is perhaps not unexpected, as cofactor binding within the pocket presumably increases PrfA DNA binding affinity via induced conformational changes that extend into the C terminal domain. It should be noted however that PrfA DNA-binding affinity does not always directly correlate with the levels of PrfA-dependent gene expression observed following PrfA activation [31], [48]. The prfA* Y63C mutation results in a highly activated form of PrfA with target gene expression levels similar to those observed for *prfA* constitutive activation mutations, such as prfA* G145S and L140F [31], [48]. However, the PrfA Y63C mutant protein binds to target promoter DNA with a similar affinity than that of wild-type PrfA [31]. The PrfA Y63 residue is also located near the putative PrfA cofactor binding site, and this mutation could potentially enhance or stabilize cofactor binding, or may augment the structural changes leading to PrfA activation that are imposed by cofactor binding. Our data, together with the *prfA* Y63C mutation, supports a model in which the individual structural domains of PrfA communicate with one another and regulate the ability of PrfA to transition into fully activated form.

In summary, our evidence indicates that the positive charge of the putative PrfA cofactor binding pocket contributes to the process of PrfA activation, and substitutions within this pocket that neutralize the positive charge reduce intracellular PrfA activity. While the identity of the cofactor remains unknown, the positive charge of the binding pocket would suggest that the cofactor is a negatively charged molecule. Modeling studies that incorporate the size and charge of the putative PrfA cofactor binding site may be useful for the identification of candidate cofactor molecules that might serve to trigger PrfA activation within host cells.

## Materials and Methods

### Ethics Statement

All animal procedures were IACUC approved by the UIC Animal Care Committee (Protocol Approval ID #09-153) and performed in the Biological Resources Laboratory at the University of Illinois at Chicago.

### Bacterial strains, plasmids and growth conditions

*L. monocytogenes* and *E. coli* strains used in this study are listed in Table S1. *E. coli* XL1-Blue (Agilent Technologies, Santa Clara, CA), One Shot TOP10 (Invitrogen Corp., Carlsbad, CA), NEB 5αF'*I^q^* (New England Biolabs, Ipswich, MA) and SM10 were used as host strains for maintenance and propagation of recombinant plasmids. *L. monocytogenes* and *E. coli* strains were grown at 37°C in brain heart infusion (BHI) media (Difco Laboratories, Detroit, MI) and Luria broth (LB) (Invitrogen Corp., Carlsbad, CA). Maintenance of the integration plasmid pPL2 [49] was selected for using 25 µg/ml of chloramphenicol in *E. coli* and 7.5 µg/ml in *L. monocytogenes*. Bacteria containing the 6X Histidine tagged expression vector pQE30 (Qiagen Inc., Valencia, CA) were maintained in *E. coli* with 100 µg/ml ampicillin. Streptomycin 200 µg/ml was used in selection of *L. monocytogenes* following bacterial conjugation and isolation from tissue organs of infected mice.

### Electrostatic Modeling

Modeling of PrfA was done using chain A of the PDB file 2BGC. The lysine to glutamine mutations were made with Coot [50]. The coordinate files were prepared for electrostatics calculations with the PDB2PQR server [51]. The electrostatic calculations were done with APBS [52] using default settings except for 0.15 mM concentrations of monovalent cations and anions. The electrostatic potentials and solvent-accessible surfaces were visualized with PyMOL [53].

### Plasmid and mutant constructions

The *prfA* lysine substitution mutations were introduced into plasmid pNF1019, a pPL2 site-specific phage integration plasmid containing a wild-type copy of *prfA* with all promoters required for expression [30], using the Change-IT Site Directed Mutagenesis Kit (USB) as per manufacture's protocol. Primers used for constructing the *prfA* lysine substitutions are listed in Table S2. The resulting plasmids were conjugated into strain NF-L1123, which contains an *actA-gus-neo* reporter gene fusion as well as an in-frame deletion of the chromosomal copy of *prfA*. For generation of purified PrfA proteins, the coding sequence of *prfA* was PCR amplified from genomic DNA of *L. monocytogenes* strains 10403S (wild-type), prfA* G145S (NF-L1226), and the pPL2 plasmids containing the various *prfA* lysine mutations using primer pairs listed in Table S2. The PCR product was then cloned into a pQE30 Expression vector (Qiagen Inc., Valencia, CA), which contains an N-terminal 6x-histidine tag and an isoproyl-β-D-thiogalactopyranoside (IPTG) inducible promoter. The resulting construct was initially propagated in *E. coli* TOP10 cells, followed by transformation into NEB 5αF'*I^q^* (New England Biolabs) for protein expression and purification. For protein expression, overnight cultures containing the expression constructs were diluted 1∶50 in fresh LB broth and incubated at 37°C (with shaking) until an optimal density of 0.5 was reached. To induce expression of the various PrfA proteins, 1 mM IPTG (Inalco, Milano, Italy) was added to the culture and induction was allowed to proceed for 3 to 4 hours. The bacterial cells were recovered by centrifugation followed by sonication with 4 repeated 10 second bursts and 1 minute cooling on ice. The soluble fraction containing the N-His-PrfA protein was collected and purified using the His-Pur Purification Kit (Thermo Scientific, Rockford, IL). Protein concentration was determined using a BCA Protein Assay Kit (Thermo Scientific, Rockford, IL).

### Western blot analysis of PrfA protein

PrfA was detected within cytoplasmic fractions isolated from bacterial whole cell extracts. 25 ml culture of each *L. monocytogenes* strain was grown to mid-log phase in BHI at 37°C with shaking. Cells were normalized to OD_600_ of 0.8, pelleted and resuspended in 1 ml of PBS. 100U of mutanolysin (Sigma-Aldrich, St. Louis, MO) was added and the suspension was incubated at 37°C for 2 hour. 10 µl of 100X Protease Inhibitor Cocktail (Calbiochem, Gibbstown, NJ) was added to the mutanolysin treated cells, followed by sonication with 3X 40 s pulses with 2 minute cooling on ice in between each pulse. Cellular debris was centrifuged at 12,000 rpm for 20 minutes at 4°C and the supernatant containing the cytoplasmic components was collected and stored at −20°C until further use. For detection of PrfA protein, 10 µl of the isolated cytoplasmic fraction was mixed with 10 µl of a 2X Laemmli Sample Buffer (BioRad Laboratories, Hercules, CA), boiled for 5 minutes, then separated by SDS-polyacrylamide gel electrophoresis. Protein samples were transferred onto PVDF membranes. PrfA was detected using a 1∶500 dilution of a monoclonal antibody directed against PrfA in 1X PBST (Phosphate buffered saline solution plus 0.05% Tween-20) followed by incubation with a 1∶2,500 dilution of a polyclonal Goat-anti Mouse secondary antibody conjugated to alkaline-phosphatase (SouthernBiotech, Birmingham, AL). Bands were visualized colorimetrically with the addition of 10 ml of a BCIP/NBT Plus solution (SouthernBiotech, Birmingham, AL). Densitometry was determined using ImageJ software (http://rsbweb.nih.gov/ij/download.html).

### Protein chemical crosslinking

Chemical crosslinking was done as previously described by Miner *et. al* with minor modifications [31]. In brief, after separation of purified protein samples by SDS-PAGE, proteins were transferred to PVDF membranes, and PrfA was detected as described above.

### Measurement of β-glucuronidase activity

Overnight cultures of *L. monocytogenes* were diluted 1∶20 in fresh BHI or LB containing 25 mM glucose-6-phosphate (Sigma-Aldrich, St. Louis, MO) and 0.2% activated charcoal and grown with shaking at 37°C. At various time points, the OD_600_ was determined for each culture and 1 ml of each sample was centrifuged. Bacterial pellets were resuspended in 1 ml of ABT buffer (1 M potassium phosphate [pH 7.0], 0.1 M NaCl, 1% Triton) and ß-Glucuronidase (GUS) activity was measured as described by Youngman [54] with the substitution of 4-methylumbelliferyl-ß-D-glucuronided (Sigma-Aldrich, St. Louis, MO) in place of 4-methylumbelliferyl-ß-D-galactoside.

### Measurement of LLO- associated hemolytic activity

Stationary-phase bacterial cultures were diluted 1∶10 into LB medium and grown at 37°C for 5 hours with shaking. Optical density OD_600_ was determined, and 1 ml of each culture was normalized and centrifuged at 13,000× g for 5 min. The supernatant was collected and was assayed for LLO-associated hemolytic activity using phosphate-buffered saline (PBS)-washed sheep erythrocytes (Cocalico Biologicals Inc., Reamstown, PA) as previously described [34]. Hemolytic activity was determined as the reciprocal of the supernatant dilution at which 50% lysis of erythrocytes was observed.

### Assessment of PlcB-associated phospholipase activity

*plcB*-dependent phospholipase production was assayed on egg yolk agar plates [48], [55]. Antibiotic-free chicken egg yolk was added in a 1∶1 (vol/vol) ratio to PBS and vortexed to form a suspension. 5 ml of egg yolk suspension was added to 100 ml of molten LB medium plus 0.2% activated charcoal (Sigma-Aldrich, St. Louis, MO) and 25 mM glucose-6-phosphate (Sigma-Aldrich, St. Louis, MO), [33], [37] and 10 ml of this mixture was poured into Petri dishes. Bacterial strains were gently streaked onto the surface of the plate and incubated at 37°C for 24 hours. Phospholipase activity was visualized as a zone of opacity surrounding bacterial streaks.

### Electrophorectic mobility shift assays (EMSAs)

Primer pairs used for amplification of the *hly* promoter DNA fragment (∼100 bp) are listed in Table S2. The 3′ end primer was purchased with a biotin label (Sigma-Aldrich, St. Louis, MO). EMSAs were done as previously described with slight modifications [31]. DNA-protein binding reactions and electrophoresis were done as described [31], protein/DNA samples were then transferred onto nylon membranes for 1 hour at a constant 0.9 amps at 4°C followed by detection of the biotinylated probe using the Pierce Chemiluminescent Nucleic Acid Detection Module (Thermo Scientific, Rockford, IL).

### Protein structural comparisons by limited proteolysis

Limited proteolytic digestion of His-purified proteins with subtilisin (Sigma-Aldrich, St. Louis, MO) was done as previously described by Miner *et al* with slight modifications [31]. In brief, 2 µg of purified protein was incubated in the presence or absence of 500 ng subtilisin for 10 and 30 minutes at room temperature followed by the addition of 1 mM phenylmethanesulfonylfluoride (PMSF) to terminate the reaction. Samples were boiled for 5 minutes and fragments were separated by Electrophoresis on a NuPAGE 4–12% Bis-Tris gel (Invitrogen Corp., Carlsbad, CA). Protein fragments were visualized by staining with Bio-Safe Coomassie G-250 (BioRad Laboratories, Hercules, CA).

### Bacterial intracellular growth assays

Bacterial intracellular growth assays in Potoroo tridactylis kidney epithelial cells (PtK2) were performed as previously described [30], [48], [55], [56]. In brief, monolayers of cells were grown on glass coverslips to confluency and infected with bacterial strains with an MOI of 100∶1. One hour post-infection, monolayers were washed 3X in PBS and 5 µg/ml of gentamicin was added to kill extracellular bacteria. At indicated time points, coverslips were removed and lysed in 5 mls of sterile H2O to release intracellular bacteria for enumeration of intracellular growth or were processed for microscopy.

### Measurement of bacterial cell-to-cell spread

Plaque assays were conducted as previously described [42]. Briefly, murine L2 fibroblasts were grown to confluency in 6-well microtiter plates and infected with 20 µl of a normalized 1∶20 dilution of overnight culture grown at 37°C in BHI with shaking (MOI 10∶1). One hour post-infection, L2 infected monolayers were washed and 5 µg/ml of gentamicin was added to kill extracellular bacteria. Three days post-infection, Neutral Red (Sigma-Aldrich, St. Louis, MO) was added and plaques were visualized and measured using a micrometer (Finescale, Orange County, CA).

### Intracellular β-glucuronidase activity

Measurement of intracellular GUS activity in PtK2 epithelial cells was done as previously described [26], [57] with minor modifications in that an MOI of 100∶1 was used and coverslips were removed at 3, 5 and 7 hours post-infection for enumeration of bacterial colony forming units.

### Mouse infections

All animal procedures were IACUC approved and performed in the Biological Resources Laboratory at the University of Illinois at Chicago. Overnight bacterial overnight cultures were diluted 1∶20 into fresh media and grown to an OD_600_ ∼0.6. 1 ml of culture (corresponding to 6×10^8^ CFU/ml) was washed, diluted and resuspended in PBS to a final concentration of 1×10^5^ CFU/ml. 8–10 week old female Swiss Webster mice (Charles River Laboratories, Chicago, IL) were injected with 200 ul PBS containing 2×10^4^ CFU *L. monocytogenes* via the tail vein. 48 hrs post-infection, mice were sacrificed and livers and spleens were harvested. Organs were homogenized with a Tissue Master 125 homogenizer (Omni International, Kennesaw, GA) and dilutions were plated onto BHI streptomycin (200 ug/ml) plates. Non-paired student *t*-test was used for statistical analysis.

## Supporting Information

Figure S1 **PlcB-associated phospholipase activity was assessed on egg yolk agar plates containing 0.2% activated charcoal and 25 mM glucose-6-phosphate following incubation at 37°C for 24 hours.** Data is representative of at least three independent experiments.
(TIF)

Table S1 **Bacterial strains and plasmids used in this study.**
(DOC)

Table S2 **Oligonucleotides used in this study.** *^a^*Letters in italicized bold indicate mutagenesis of lysine (AAA) to glutamine (CAG) or of glycine (GGT) to serine (AGT). Letters in bold indicate the second and stop codons of the *prfA* coding sequence. Italicized letters indicate the *Kpn*I and *Pst*I restriction endonuclease sites on the forward and reverse primers used for cloning the PCR fragment into pQE30 expression vector.
(DOC)
