# Supplementary figures and images for: Probing the Role of Protein Surface Charge in the Activation of PrfA, the Central Regulator of Listeria monocytogenes Pathogenesis
Source: PLoS One. 2011 Aug 12;6(8):e23502. doi: 10.1371/journal.pone.0023502 (PMC3155570; doi:10.1371/journal.pone.0023502)

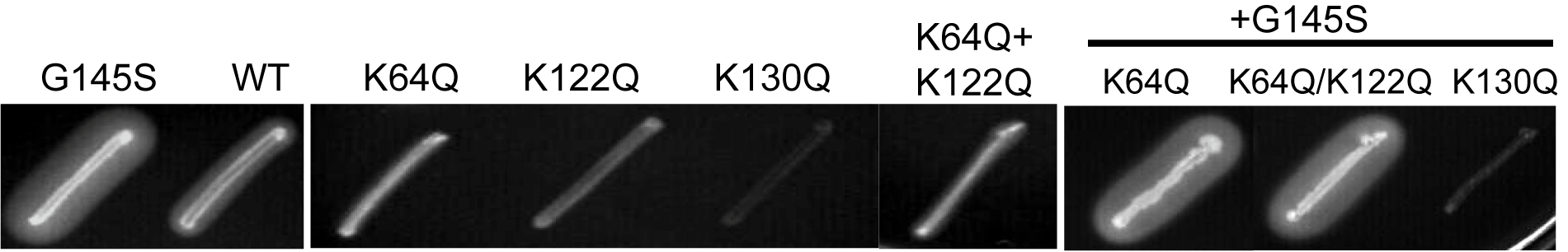

Supplement: Figure S1 — PlcB-associated phospholipase activity was assessed on egg yolk agar plates containing 0.2% activated charcoal and 25 mM glucose-6-phosphate following incubation at 37°C for 24 hours. Data is representative of at least three independent experiments. (TIF) [file pone.0023502.s001.tif]
