# Supplementary material for: Probing the Role of Protein Surface Charge in the Activation of PrfA, the Central Regulator of Listeria monocytogenes Pathogenesis
Source: PLoS One. 2011 Aug 12;6(8):e23502. doi: 10.1371/journal.pone.0023502 (PMC3155570; doi:10.1371/journal.pone.0023502)
Supplement: Table S1 — Bacterial strains and plasmids used in this study. (DOC) [file pone.0023502.s002.doc]

**Table S1**. Bacterial strains and plasmids used in this study.

| **Strain** | **Description** | **Source/Reference** |
| --- | --- | --- |
| ***L. monocytogenes*** |  |  |
| NF-L100 | Wild type 10403S | [1,2] |
| NF-L1003 | Δ*prfA* *actA-gus-plcB* fusion | [3] |
| NF-L1041 | pPL2-WT *prfA* (pNF-1019) in Δ*prfA* strain NF-L1003 | [3] |
| NF-L1123 | Δ*prfA* *actA-gus-neo-plcB* fusion | [4] |
| NF-L1226 | pPL2- *prfA* G145S in Δ*prfA* strain NF-L1003 | [5] |
| NF-L1917 | pNF-1716 (K130Q) in NF-L1123 | This study |
| NF-L1920 | pNF-1719 (K64Q) in NF-L1123 | This study |
| NF-L2039 | pNF-1717 (K122Q) in NF-L1123 | This study |
| NF-L2078 | pNF-2068 (K64Q/K122Q) in NF-L1123 | This study |
| NF-L3030 | pNF-3022 (K64Q+G145S) in NF-L1123 | This study |
| NF-L3031 | pNF-3023 (K130Q+G145S) in NF-L1123 | This study |
| NF-L3032 | pNF-3024 (K64Q/K122Q+G145S) in NF-L1123 | This study |
| ***E. coli*** |  |  |
| XL1-Blue | *E. coli* propagation strain | Agilent Technologies |
| NEB 5αF’I*^q^* | *E. coli* protein expression strain | NEB |
| TOP10 | *E. coli* propagation strain | Invitrogen |
| SM10 | *E. coli* conjugation strain |  |
| **Plasmids** |  |  |
| pQE30 | N-terminal His-tagged expression vector | Qiagen |
| pNF-1019 | pPL2 site specific integration vector with full length *prfA* and all promoters | [3] |
| pNF-1716 | pNF-1019 *prfA* K130Q | This study |
| pNF-1717 | pNF-1019 *prfA* K122Q | This study |
| pNF-1719 | pNF-1019 *prfA* K64Q | This study |
| pNF-2036 | pQE30 *prfA* K64Q | This study |
| pNF-2037 | pQE30 *prfA* K122Q | This study |
| pNF-L2038 | pQE30 *prfA* K130Q | This study |
| pNF-2068 | pNF-1019 *prfA* K64Q/K122Q | This study |
| pNF-2075 | pQE30 *prfA* K64Q/K122Q | This study |
| pNF-2076 | pQE30 *prfA* wild-type | This study |
| pNF-2077 | pQE30 *prfA* L140F | This study |
| pNF-3022 | pNF-1019 *prfA* K64Q+G145S | This study |
| pNF-3023 | pNF-1019 *prfA* K130Q+G145S | This study |
| pNF-3024 | pNF-1019 *prfA* K64Q/K122Q+G145S | This study |
| pNF-3025 | pQE30 *prfA* K64Q+G145S | This study |
| pNF-3026 | pQE30 *prfA* K130Q+G145S | This study |
| pNF-3027 | pQE30 *prfA* K64Q/K122Q+G145S | This study |
| pNF-3028 | pQE30 *prfA* G145S | This study |

**Supplemental References for Table S1.**

1. Bishop DK, Hinrichs DJ (1987) Adoptive transfer of immunity to *Listeria monocytogenes*. The influence of in vitro stimulation on lymphocyte subset requirements. J Immunol 139: 2005-2009.

2. Edman DC, Pollock MB, Hall ER (1968) *Listeria monocytogenes* L forms. I. Induction maintenance, and biological characteristics. J Bacteriol 96: 352-357.

3. Wong KK, Freitag NE (2004) A novel mutation within the central *Listeria monocytogenes* regulator PrfA that results in constitutive expression of virulence gene products. J Bacteriol 186: 6265-6276.

4. Miner MD, Port GC, Bouwer HG, Chang JC, Freitag NE (2008) A novel *prfA* mutation that promotes *Listeria monocytogenes* cytosol entry but reduces bacterial spread and cytotoxicity. Microb Pathog 45: 273-281.

5. Port GC, Freitag NE (2007) Identification of novel *Listeria monocytogenes* secreted virulence factors following mutational activation of the central virulence regulator, PrfA. Infect Immun 75: 5886-5897.
