# Supplementary material for: Probing the Role of Protein Surface Charge in the Activation of PrfA, the Central Regulator of Listeria monocytogenes Pathogenesis
Source: PLoS One. 2011 Aug 12;6(8):e23502. doi: 10.1371/journal.pone.0023502 (PMC3155570; doi:10.1371/journal.pone.0023502)
Supplement: Table S2 — Oligonucleotides used in this study. aLetters in italicized bold indicate mutagenesis of lysine (AAA) to glutamine (CAG) or of glycine (GGT) to serine (AGT). Letters in bold indicate the second and stop codons of the prfA coding sequence. Italicized letters indicate the KpnI and PstI restriction endonuclease sites on the forward and reverse primers used for cloning the PCR fragment into pQE30 expression vector. (DOC) [file pone.0023502.s003.doc]

**Table S2.** Oligonucleotides used in this study.

| **Primer** | **Sequence (5’→3’)*^a^*** | **Reference^b^** |
| --- | --- | --- |
| K64Q | GGACCATCATGAATTTACAATACTAC***CAG***GGGGCTTTCGTTATAATGTCGGC | This study |
| K122Q | TTTCTATGTTTTCCAAACCCTACAA***CAG***CAAGTTTCATACAGCCTAGCTAATT | This study |
| K130Q | AAACAAGTTTCATACAGCCTAGCT***CAG***TTTAATGATTTTTCGATTAACGGGA | This study |
| G145S | TTAACGGGAAGCTTGGCTCTATTTGC***AGT***CAACTTTTAATCCTGACCTATGTGT | This study |
| pPL2-common | AAGTAGATGCTTTTTTCACACTAAAAACAGATGAAAATACCACAATT | This study |
| HisPrfA-F | AAA*GGTACC***AAC**GCTCAAGCAGAAG | This study |
| HisPrfA-R | GG*CTGCAG*T**TTA**ATTTAATTTTCCCCAAG | This study |
| 5’hly | TCCTATCTTAAAGTGACTTTATGTT | [1] |
| 3’hly | GCTTCTAAAGATGAAACGCAATATTA | [1] |

**Supplemental Reference for Table S2.**

1. Miner MD, Port GC, Freitag NE (2008) Functional impact of mutational activation on the *Listeria monocytogenes* central virulence regulator PrfA. Microbiology 154: 3579-3589.
